# Evaluation of the Effect of Different Treatment Management on Refractive Outcomes in Severe Retinopathy of Prematurity

**DOI:** 10.14744/SEMB.2021.34966

**Published:** 2021-12-29

**Authors:** Semra Tiryaki Demir, Sumeyra Keles Yesiltas, Murat Karapapak, Emine Betul Akbas Ozyurek, Ali Bulbul, Hasan Sinan Uslu, Dilek Guven

**Affiliations:** 1. Department of Ophthalmology, University of Health Sciences Turkey, Prof Dr Cemil Tascioglu City Hospital, Istanbul, Turkey; 2. Department of Ophthalmology, University of Health Sciences Turkey, Sisli Hamidiye Etfal Training and Research Hospital, Istanbul, Turkey; 3. Department of Ophthalmology, University of Health Sciences Turkey, Basaksehir Cam and Sakura City Hospital, Istanbul, Turkey; 4. Department of Neonatology, University of Health Sciences Turkey, Sisli Hamidiye Etfal Training and Research Hospital, Istanbul, Turkey

**Keywords:** Anisometropia, intravitreal bevacizumab, laser photocoagulation, myopia; refraction

## Abstract

**Objectives:**

The purpose of the study was to evaluate the effect of different treatment modalities on refractive outcomes in patients treated with severe retinopathy of prematurity (ROP).

**Methods:**

The records of children who were treated for severe ROP in our clinic between January 2015 and August 2018 were retrospectively reviewed. The children who were treated were analyzed in three subgroups as intravitreal bevacizumab (IVB), laser photocoagulation (LPC), and IVB + LPC. Spherical equivalent (SEQ), spherical and cylindrical power measurements of the cases were recorded in diopters (D). SE ≤−0.25D was accepted as myopia and SE of more than 1 D between two eyes was accepted as anisometropia.

**Results:**

A total of 160 eyes of 80 participants were eligible for inclusion: 38 eyes in the IVB group, 24 eyes in the LPC group, 16 eyes in the IVB + LPC group, 44 eyes in the spontaneously regressed group, and 38 eyes in the full-term children. Although the mean spherical power and SEQ in the IVB group were lower than in the LPC group (p=0.019 and 0.013, respectively), there was no significant difference between the IVB group and the IVB + LPC group (p=0.541 and 0.804, respectively). In terms of mean cylindrical power and prevalence of myopia and anisometropia, there was no significant difference between the treatment groups (p>0.05).

**Conclusion:**

Although spherical power and SEQ can change according to the ROP treatment management, there is no difference in terms of the cylindrical power, prevalence of myopia, and anisometropia. The most important risk factor for myopia and anisometropia in premature children may be ROP severity and retinal immaturity.

Retinopathy of prematurity (ROP) was first described in the year 1942 by Terry.^[1]^ If it is not intervened in time, it results in serious vision loss.^[2]^ With the development and widespread use of neonatal intensive care units in recent years, the number of premature babies that can be kept alive and ROP incidence has been increasing.^[2]^ The most strongest risk factors in the development of ROP are gestational age (GA) and low birth weight (BW). Ablation of the entire peripheral avascular retina with laser photocoagulation (LPC) is the gold standard treatment for ROP. Today, antivascular endothelial growth factor agents (anti-VEGF) are also commonly used in the treatment of ROP.^[3]^ Both treatments have been shown to be safe and effective in the treatment of severe ROP.^[4]^

There are studies evaluating the refractive outcome in the advanced ages of premature infants with or without severe ROP.^[5-7]^ Refractive problems such as anisometropia, myopia, and astigmatism have been shown widely in children who were born prematurely.^[5]^ There are studies evaluating the effect of LPC and anti-VEGF treatments on refractive outcomes.^[8,9]^

In this study, we aimed to assess the effect of different treatment modalities on refractive outcomes in children where we have previously applied ROP treatment. We examined spherical power, cylindrical power, spherical equivalent (SEQ), and myopia and anisometropia prevalence in these children. In addition, we also compared refractive outcomes of these children with children with spontaneously regressed ROP and full-term children.

## Methods

This study was approved by the local human research Ethics Committee, in accordance with the Declaration of Helsinki, and written informed consent was obtained from all participants’ parents or guardians (2654/2020).

In this retrospective, non-randomized, cross-sectional, observational clinical study that included patients aged 18 months–5 years who underwent ROP treatment in our clinic between January 2015 and August 2018. It was compared with full-term children and children with spontaneously regressed who were prematurely born and did not need ROP treatment (Stage 1 or Stage 2 ROP). BW, GA, gender, and chronological age during the examination of all participants were recorded.

Inclusion criteria are as follows: (1) Follow-up of at least 18 months, (2) GA ≤34 weeks,^[10]^ (3) the refractive media to allow successful retinoscopy, (4) the child ensures accurate fixation the light target and maintains a stable head position during the process, and (5) there was no other organic eye disease other than ROP. Exclusion criteria are as follows: (1) GA ≥35 weeks,^[10]^ (2) follow-up <18 months, (3) neurologic abnormalities such as ischemic pathologies and cranial nerve palsies (neurologic abnormalities are diagnosed by pediatric neurology consultation), (4) macular dragging, Stage 4 or 5 ROP, (5) a clear refractive media opacity and preventing retinoscopy and detailed imaging, (6) children who were unable to cooperate during the examination, and (7) the existence of ocular disease other than ROP or systemic disease.

As stated in the criteria set by the American Academy of Pediatrics, the American Association of Pediatric Ophthalmology and Strabismus, American Academy of Ophthalmology premature infants with GA of <30 weeks, BW <1500 g or GA of >30 weeks but whose clinical course was not stable were examined for ROP.^[11]^ According to the criteria set by the International ROP Committee, the zones and stages of the cases were recorded.^[12]^ According to the Early Treatment for ROP study, patients with type 1 ROP and aggressive posterior ROP (APROP) were treated.^[13]^ All treatments were done by two ophthalmologists (STD and DG).

As the first treatment option, intravitreal bevacizumab (IVB) treatment was applied to patients with Zone 1 and APROP, while conventional diode LPC treatment was applied to patients with Zone 2 ROP. Furthermore, IVB treatment was applied to patients with Zone 2 ROP whose pupil is not dilated and has intravitreal bleeding that does not allow LPC treatment. LPC treatment was applied to patients who developed recurrence or inadequate response after IVB treatment (IVB + LPC). The patients who were treated were analyzed in three subgroups as IVB group, LPC group, and IVB + LPC group, and compared with spontaneously regressed and full-term children.

### IVB Treatment

A lid speculum was inserted in the eye. After topical or sedation anesthesia with 0.5 mg% proparacaine and administration of 10% povidone antiseptic solution to the ocular surface, 0.625 mg/0.025 mL bevacizumab (Altuzan®, Roche, Turkey) was given into the vitreous with a 30-gauge needle. The injection was applied 1.5 mm behind the limbus and through the pars plicata.

### LPC Treatment

The LPC treatment was applied under general or sedation anesthesia. An indirect laser with a wavelength of 810 nm (Iridex, OcuLight SL, USA) was then used to apply photocoagulation to the entire avascular retina in a nearly confluent fashion.

### Refractive Error Assessment

Cycloplegic refraction was recorded by the Spot Vision handheld autorefractometer (Spot; Welch Allyn, Skaneateles Falls, New York, USA) after three drops of cyclopentolate HCl 1% were administered 5 min apart at the final visit. To find consistency between measurements, refraction findings were measured 3 times. In case of non-compliance in these measurements, measurements were repeated until three consecutive compatible measurements were detected. Measured refraction errors were checked by retinoscopy. Refraction measurement was tested before the fundus examination. The SE was determined by adding together the spherical value and half of the cylindrical value. Anisometropia was defined as SE of more than 1 D between two eyes, myopia was defined as SE of ≥0.25 D of myopia, and high myopia was defined as a SE of ≥5.00 D of myopia.^[14,15]^

### Statistical Analysis

Mean, standard deviation, and ratio values were used in descriptive statistics of the data. Kruskal–Wallis test was used for the analysis of quantitative independent data. Chi-square test was used to analyze independent data. Non-parametric post-hoc test was used to evaluate whether there was a difference between which groups. Statistical Package for the Social Sciences 22.0 program was used in the analyzes.

## Results

A total of 160 eyes of 80 participants were eligible for inclusion: 38 eyes in the IVB group, 24 eyes in the LPC group, 16 eyes in the IVB + LPC group, 44 eyes in the spontaneously regressed group, and 38 eyes in the control group. The distribution of GA, BW, chronological age, gender, spherical power, cylindrical power, and SEQ of all participants is shown in Table 1. The mean GA and BW were significantly different between the groups (p<0.001). The mean GA and BW were not significantly different between treatment groups and spontaneously regressed group (p>0.05). There was no significant difference between the gender distributions and mean chronological age between the groups (respectively, p=0.325 and 0.142).

**Table 1.** Distribution of gestational age, BW, postmenstrual age, gender, spherical values, cylindrical value, and spherical equivalent of all participants

	**IVB group (n=19), (23.7%) mean±SD**	**LPC group (n=12), (15%) mean±SD**	**IVB+LPC group (n=8), (9.8%) mean±SD**	**Spontaneous regression group (n=22), (27.5%) mean±SD**	**Control group (n=19), (23.7%) mean±SD**	**p**
GA, wk	29.4±2.5	29±2.9	27.7±3.3	30.8±2.6	39.3±0.9	<0.001 K
BW, g	1327±393	1447±578	1158±512	1548±462	3291±424	<0.001 K
Chronological age, m	33.05±13.41	38.08±14.59	36.88±18.71	27.82±7.24	28.21±11.58	0.142 K
Gender						
Female/male	10/9	8/4	5/3	8/14	7/12	0.325 X2
Spherical value, D	1.28±2.06	2.67±2.25	1.56±2.27	2.33±1.03	2.28±1.26	0.028 K
Cylindrical value, D	1.68±0.76	1.13±0.75	1.42±0.82	0.90±0.67	1.04±0.64	<0.001 K
Spherical equivalent, D	0.46±1.93	2.10±2.24	0.88±2.31	1,91±1.05	1,78±1.12	<0.001 K

K: Kruskal–Wallis test; χ2: Chi-square test; IVB: Intravitreal bevacizumab; LPC: Laser photocoagulation; SD: Standard deviation; GA: Gestational age; wk: Weeks; BW: Birth weight; g: Gram; m: Month; D: Diopter.

**Figure 1.**
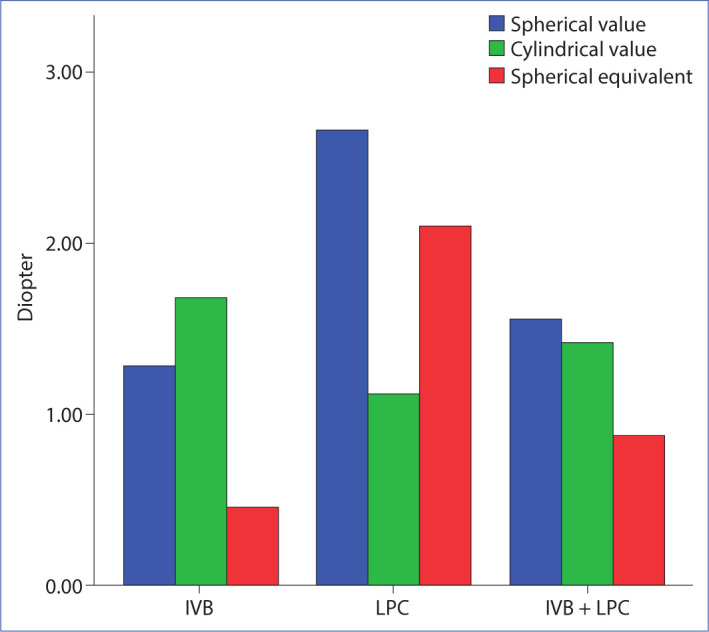
Graphical representation of the mean spherical values, cylindrical values, and spherical equivalents of the treated groups. IVB: Intravitreal bevacizumab, LPC: Laser photocoagulation. One-way analysis of variance test: Spherical power, cylindrical power, spherical equivalent, respectively, p=0.027, 0.062, 0.014. Post-hoc test: Spherical power IVB versus LPC P=0.019, IVB versus IVB+LPC p=0.541, LPC versus IVB+LPC p=0.486. Spherical equivalent IVB versus LPC p=0.013, IVB versus IVB+LPC p=0.804, LPC versus IVB+LPC p=0.232.

The mean spherical power, the cylindrical power, and the SEQ of the treatment groups are shown in Figure 1. The mean spherical power was significantly different between the treatment groups (p=0.027). In the treatment groups, mean spherical power from the lowest to the highest one was as follows: IVB group, IVB + LPC group, and LPC group. Although the mean spherical power in the IVB group was significantly lower than the LPC group (p=0.019), there was no significant difference between IVB group and IVB + LPC group (p=0.541).

The mean cylindrical power was not significantly different between the treatment groups (p=0.062). In the treatment groups, mean cylindrical power from the highest to the lowest one was as follows: IVB group, IVB + LPC group, and LPC group. The mean SEQ was significantly different between the treatment groups (p=0.014). In the treatment groups, mean SEQ from the lowest to the highest one was as follows: IVB group, IVB + LPC group, and LPC group. Although the mean SEQ in the IVB group was significantly lower than the LPC group (p=0.013), there was no significant difference between IVB group and IVB + LPC group (p=0.804).

Myopia was detected in 17 eyes (10.6%) among all participants. Myopia was present in 7 eyes (18.4%) in the IVB group, 3 eyes (12.5%) in the LPC group, 6 eyes (37.5%) in the IVB + LPC group, and 1 eye (2.2%) in the spontaneously regressed group. In the treatment groups, prevalence of myopia from the highest to the lowest one was as follows: IVB + LPC group, IVB group, and LPC group. In terms of prevalence of myopia, there was no significant difference between the treatment groups (p=0.195). In terms of myopia prevalence, although there was a significant difference between treatment groups and spontaneously regressed group (p<0.001), there was no significant difference between the spontaneously regressed group and the control group (p=0.212). High myopia was detected in only one eye of the patient in the IVB group (Zone 1 APROP). The SEQ of the right eye was –6.00, the SEQ of the left eye was –1.75, and anisometropia was present between the two eyes.

Anisometropia was detected in 7 cases (8.7%) among all participants. Anisometropia was present in 3 cases (15.8%) in the IVB group, 2 cases (16.6%) in the LPC group, and 2 cases (25%) in the IVB + LPC group. In the treatment groups, prevalence of anisometropia from the highest to the lowest one was as follows: IVB + LPC group, LPC group, and IVB group. Anisometropia was not found in the spontaneously regressed group and control group. In terms of prevalence of anisometropia, there was no significant difference between the treatment groups (p=0.867).

## Discussion

This study examining refractive outcome of children between 18 months and 5 years old who underwent any ROP treatment. Children who underwent any ROP treatment were compared with children with per se regressed and full-term children. Even though there is a significant difference among treatment groups in terms of spherical power and SEQ, there was no significant difference in the pervasiveness of anisometropic, cylindrical power, and myopia. In terms of the prevalence of anisometropia and myopia, although children who had undergone any ROP treatment were significantly higher than those who were children with spontaneously regressed and full-term children, no significant difference was detected between the different ROP treatment managements.

Refraction measurements in premature infants in groups with and without ROP who received treatment or untreated were investigated.^[16]^ It was declared that refraction at 2.5 years may be a better estimator of refractive measurement in older ages.^[6]^ The relationship between ROP treatment managements and spherical power, cylindrical power, and SEQ has been examined and different results have been reported. Mueller et al.^[9]^ demonstrated that SEQ was not significantly different between IVB and LPC in posterior ROP patients, and lower SEQ in infants with posterior ROP compared with peripheral Zone II. Gunay et al.^[17]^ stated that SEQ with Zone I ROP was more myopic than that with Zone II ROP. Kang et al.^[18]^ reported no significant differences in spherical power, cylinder power, and SEQ between laser-treated eyes or anti-VEGF-treated eyes. On the contrary, Roohipoor et al.^[19]^ reported that spherical power and SEQ were significantly higher in eyes treated with LPC than eyes treated with IVB, no significant difference between LPC and IVB treatment in terms of astigmatic power. Although astigmatism remains a major factor in most children treated with IVB, it is milder than children treated with laser.^[20,21]^ Unlike other publications, Kabataş et al.^[22]^ observed that astigmatism powers were akin with all treated and untreated groups.

In our study, although spherical power and SEQ were significantly lower than LPC-treated eyes in IVB-treated eyes, there was no significant difference between IVB and IVB + LPC-treated eyes. Cylindrical power was higher in eyes treated with spontaneous regression. There was no significant difference in terms of cylindrical power between the treatment groups.

It has been shown before that the refractive state is different between mature infants and premature infants, and those who receive ROP treatment have a higher chance of developing myopia, astigmatism, and higher diopters.^[16]^ Mintz-Hittner et al.^[23]^ reported that myopia seen in the face of ROP and prematurity is multifactor in etiology, with three main causative factors: (1) Prematurity, (2) the severity of ROP, and (3) different treatment options for ROP. Thicker lenses, vertical, steeper corneal curvature, and shallower anterior chamber depth are the main factors contributing to the progress of myopia in children with laser-treated ROP.^[24]^ However, IVB therapy is thought to be able to continue ocular growth factor expression leading to decreased myopia and anterior segment development in its normal course.^[8]^ In the bevacizumab eliminates the angiogenic threat ROP study group, refractive results following IVB treatment in infants with an average age of 2.5 years were evaluated. This study showed lower prevalence of myopia following IVB therapy compared to LPC therapy, and eyes with Zone II ROP are significantly less myopic compared with eyes with Zone I ROP.^[8]^ Tan et al.^[20]^ evaluated progress of refractive fault in children treated for severe ROP by anti-VEGF agents with a meta-analysis study. They showed that pervasiveness of myopia remains high after IVB treatment, and compared with laser-treated group, IVB-treated group has less myopic refractive value, lower pervasiveness of high myopic measurement, and less astigmatism. Chen et al.^[25]^ compared IVB monotherapy group with IVB + LPC group due to recurrence or inadequate treatment. They indicated that the frequency of myopic refractive error was higher in those treated with IVB + LFK compared to treated with IVB monotherapy at 2 years of age. Unlike other publications, Kuo et al.^[26]^ reported that myopic value between laser and IVB treatment in patients with type 1 ROP was not statistically different. Requiring treatment ROP eyes are vulnerable to more serious myopic measurement with age compared with eyes without ROP or those with naturally regressed ROP. Isaac et al.^[27]^ reported that both IVB and LPC treatments resulted no difference in refraction.

In our study, IVB and IVB + LPC treatment was implemented to eyes with APROP and Zone 1 ROP, and LPC treatment was mostly implemented to eyes with Zone 2 ROP. That is, IVB and IVB + LPC treatment was applied to the eyes with more immature retina. Although myopia prevalence was higher in eyes treated with IVB + LPC, no significant difference was detected between treatment groups. Myopia prevalence was significantly higher in treated eyes than in spontaneously regressed eyes. There was no difference between full term and spontaneously regressed. Therefore, we think that the most critical risk factor for myopia seen in premature children is the severity of the ROP and retinal immaturity.

Anisometropia demonstrates higher risk of amblyopia at an early age in this population.^[20]^ Anisometropia has been shown to develop more often in severe cases of ROP who received treatment.^[7]^ As reported by Gunay et al.,^[28]^ incidence of anisometropia was observed more frequently in the laser-treated group than in the IVB group.

In our study, anisometropia was detected only in the treated eyes, but not in the spontaneously regressed eyes. Although anisometropia prevalence was higher in eyes treated with IVB + LPC, no significant difference was detected between treatment groups. Hence, we think that the most critical risk factor for anisometropia seen in premature children is ROP treatment requirement and retinal immaturity. Our study has some strengths. We evaluated the all participants in five subgroups as IVB, LPC, IVB + LPC, spontaneously regressed, and full term. We analyzed the patients who developed recurrence or inadequate response after IVB treatment under a separate group as IVB + LPC. No significant difference was detected between treatment groups and the spontaneously regressed group in terms of BW, GA, and chronological age, which may affect refractive outcomes. We also evaluated anisometropia, which can cause amblyopia. There are still some limitations in our study. These include the retrospective design of the study, the small case population, and the lack of biometric tests for instance anterior chamber depth and axial length.

## Conclusion

Refractive outcomes of pre-terms who spontaneously regressed or developed severe ROP differ in childhood. Refractive errors such as anisometropia, myopia, or astigmatism that can develop in childhood are particularly associated with the severity of ROP and retinal immaturity rather than prematurity or ROP treatment managements. It is appropriate to periodically perform refraction examinations to detect refractive errors that may require optical correction in children who have undergone any ROP treatment due to severe ROP. Further studies involving more patients, especially prospective long follow-up trials, are necessary to better define which patient will benefit most from each treatment modality.

### Disclosures

**Ethics Committee Approval:** This study was approved by the local human research Ethics Committee, in accordance with the Declaration of Helsinki, and written informed consent was obtained from all participants’ parents or guardians (2654/2020).

**Peer-review:** Externally peer-reviewed.

**Conflict of Interest:** None declared.

**Authorship Contributions:** Concept – S.T.D.; Design – S.T.D.; Supervision – A.B., D.G., H.S.U.; Materials – S.T.D.; Data collection/processing – S.K.Y., M.K., E.B.A.O.; Analysis and/or interpretation – S.T.D.; Literature search – S.T.D., S.K.Y., M.K., E.B.A.O.; Writing – S.T.D.; Critical review – A.B., D.G., H.S.U.
